# Synthetic Sweeteners and Human Health: An Overview of Health Risks, Vulnerable Populations, and Effects on Key Biological Systems

**DOI:** 10.3390/medicina62061138

**Published:** 2026-06-11

**Authors:** Stanislava Ivanova, Stanislav Dyankov, Vanya Nalbantova, Michaela Shishmanova-Doseva, Iva Slavova, Kremena Saracheva

**Affiliations:** 1 Department of Pharmacognosy and Pharmaceutical Chemistry, Faculty of Pharmacy, Medical University of Plovdiv, 4002 Plovdiv, Bulgaria; 2 Research Institute, Medical University of Plovdiv, 4002 Plovdiv, Bulgaria; 3 PERIMED-2, BG16RFPR002-1.014-0007, Central District, Vasil Aprilov Blvd. 15A, 4002 Plovdiv, Bulgaria; 4 Department of Pharmacology, Toxicology and Pharmacotherapy, Faculty of Pharmacy, Medical University of Plovdiv, 4002 Plovdiv, Bulgaria; 5 Department of Chemical Sciences, Faculty of Pharmacy, Medical University of Plovdiv, 4002 Plovdiv, Bulgaria

**Keywords:** acesulfame, advantame, artificial sweeteners, aspartame, cyclamate, neotame, public health, saccharin, sucralose, sugar

## Abstract

*Background and Objectives*: Nowadays synthetic sweeteners are widely used as sugar substitutes in beverages, processed foods, and pharmaceutical products, largely due to their low caloric content and perceived benefits for weight management and glycemic control. Their consumption has increased markedly over recent decades, paralleling global efforts to reduce added sugar intake and combat obesity and diabetes. This review examines the regulation of artificial sweeteners, their impact on vulnerable populations, and the increased concern about their health effects, including metabolic effects, effects on gut microbiota and neurological and behavioral issues. *Materials and Methods*: A comprehensive search was performed across multiple electronic databases, including PubMed, Scopus, Web of Science, and Google Scholar, to identify studies relevant to synthetic sweeteners and human health. *Results*: While considered safe, artificial sweeteners are linked to potential influence on hormonal responses, affecting glucose homeostasis and insulin secretion, as well as effects on gut microbiota composition and glucose metabolism. However, the results reveal inconsistencies of the impact of artificial sweeteners on vulnerable populations, as well as their effects on the human gut microbiota, neurological behavior and endocrine effects and evidence remain limited. *Conclusions*: Continuous human trials, post-market surveillance and regulatory evaluations are therefore essential to ensure the safety of sugar substitutes for consumers’ health.

## 1. Introduction

Nowadays, synthetic sweeteners are widely used as sugar substitutes in beverages, processed foods, and pharmaceutical products, largely due to their low caloric content and perceived benefits for weight management and glycemic control [1]. Their consumption has increased markedly over recent decades, paralleling global efforts to reduce added sugar intake and combat obesity and diabetes [2,3]. The global artificial sweetener market is substantial and continues to expand. Recent market analyses estimate the global artificial sweetener market at approximately USD 4.28 billion in 2026, with a projected value of USD 5.38 billion by 2031, reflecting a compound annual growth rate (CAGR) of 4.36% [4]. In contrast, another market assessment reports a higher valuation of USD 10.8 billion in 2026, with an expected increase to USD 15.6 billion by 2036 at a CAGR of 3.8% [5]. Despite this growing demand, the commercialization of artificial sweeteners is constrained by persistent concerns regarding their safety and potential health effects, as numerous studies have associated their consumption with risks such as cancer, obesity, and other adverse health outcomes [6,7].

The development of artificial sweeteners dates to the late nineteenth century, with the discovery of saccharin in 1879 by Constantin Fahlberg. Subsequently, cyclamate (1937), aspartame and acesulfame potassium (1967), sucralose (1970s), neotame (1990s), and advantame (2000s) were introduced, expanding the range of available sweeteners for commercial use [8]. These sweeteners are most frequently incorporated into sugar-free beverages and dairy products, confectionery, baked goods, puddings, and used as tabletop sweeteners [9]. In addition, artificial sweeteners are widely employed in pharmaceutical formulations as excipients to mask the bitter or unpleasant taste of active ingredients, including in syrups, chewable tablets, lozenges, and orally disintegrating dosage forms [10]. Their widespread use is particularly evident in regions with high consumption of processed foods and diet beverages, including North America, Europe, China, Japan, and several countries in Latin America, while countries in the Asia-Pacific region, particularly China, India, and Japan, dominate the global market [4].

Among the currently approved artificial sweeteners, sucralose, aspartame, saccharin, acesulfame potassium, neotame, and advantame are the most widely used worldwide [1,9]. These sweeteners differ in their chemical structure, which influences their degree of sweetness and metabolism in the human body [11]. For instance, aspartame, a methyl ester of a dipeptide, consisting of phenylalanine and aspartic acid, is metabolized completely via hydrolysis after ingestion [12,13]. In contrast, the more metabolically stable sulfonamide derivatives saccharin and acesulfame-K are subject to minimal metabolism and are excreted mostly unchanged [14,15]. Furthermore, newer sweeteners such as neotame and advantame are characterized by significantly higher sweetness intensity, which leads to reduced dietary exposure [16,17]. These differences are important because they may influence the physiological effects and the safety profiles of these substances [18].

Despite regulatory approval and extensive use, the safety of synthetic sweeteners remains a subject of ongoing scientific debate [19]. Emerging evidence has raised concerns regarding potential adverse effects on metabolic health, gut microbiota, neurological function, and long-term disease risk [20,21]. At the same time, results from different studies are often inconsistent, complicated by differences in study design, exposure levels, and funding sources [18,19].

This review examines evidence on the health risks associated with synthetic sweeteners, with attention to areas of controversy, vulnerable populations, and limitations in existing research. The aim is to provide an assessment that informs both scientific understanding and public health decision-making.

## 2. Materials and Methods

The literature selection process was performed across multiple electronic databases, including PubMed, Scopus, Web of Science, and Google Scholar, to identify studies relevant to synthetic sweeteners and human health. A broad set of keywords was used to maximize retrieval of relevant publications. Search terms included “synthetic sweeteners”, “non-nutritive sweeteners”, “artificial sweeteners”, “aspartame”, “sucralose”, “saccharin”, “acesulfame potassium”, “neotame”, “advantame”, “cyclamate”, “metabolic effects”, “gut microbiota”, “neurological effects”, “endocrine effects”, and “human health”. The search included articles published up to May 2026. A total of 16,075 records were initially identified across all databases.

After removal of duplicates, titles and abstracts were screened for relevance. Full-text articles were subsequently assessed. Studies were included if they: (1) were peer-reviewed original articles; (2) investigated synthetic or non-nutritive sweeteners; and (3) evaluated metabolic, endocrine, neurological, behavioral, gastrointestinal or gut microbiota-related effects in humans or relevant animal models. Animal studies were included only when they provided important mechanistic insights relevant to human health. Studies were excluded if they: (1) were non-peer-reviewed publications; (2) lacked sufficient methodological details or outcome data; or (3) were unrelated to the health effects of synthetic sweeteners regarded in this review. Following the screening process, eighty-six original studies were included in the final review, with sixty-six studies reflecting vulnerable populations and metabolic/endocrine/neurological/behavioral/gastrointestinal/gut microbiota effects in humans or relevant animal models, and twenty studies included for broader context and supporting evidence.

## 3. Results and Discussion

### 3.1. Overview of Synthetic Sweeteners

#### 3.1.1. Aspartame

**Figure 1:**
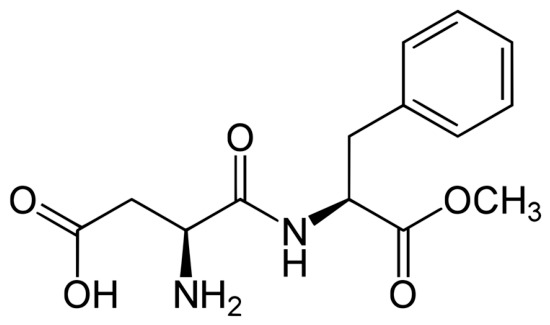
Chemical structure of aspartame.

Aspartame (Figure 1) is a synthetic, low-calorie sweetener belonging to the class of dipeptide methyl esters [22]. Chemically, it is composed of two naturally occurring amino acids, L-aspartic acid and L-phenylalanine, linked by a peptide bond, with a methyl ester group attached to the carboxyl group of phenylalanine [23,24]. Its molecular formula is C_14_H_18_N_2_O_5_, and it has a molecular weight of approximately 294.3 g/mol [23]. It was discovered in 1965 and first approved by the United States Food and Drug Administration (FDA) in 1974 [9].

Aspartame is about 200 times sweeter than sucrose, allowing its use in very small quantities [9]. The sweetness is stereospecific and depends on the L-configuration of both amino acids. The L-L stereoisomer provides sweetness, while alteration of stereochemistry reduces sweet taste and induces bitterness [25]. From a chemical standpoint, aspartame is relatively unstable under high temperatures and extreme pH conditions, undergoing hydrolysis that leads to loss of sweetness, in addition to possible photolytic degradation [24,26]. For this reason, it is primarily used in cold or shelf-stable products such as soft drinks, tabletop sweeteners, and dairy-based foods, rather than in baked goods [27].

**Figure 2:**
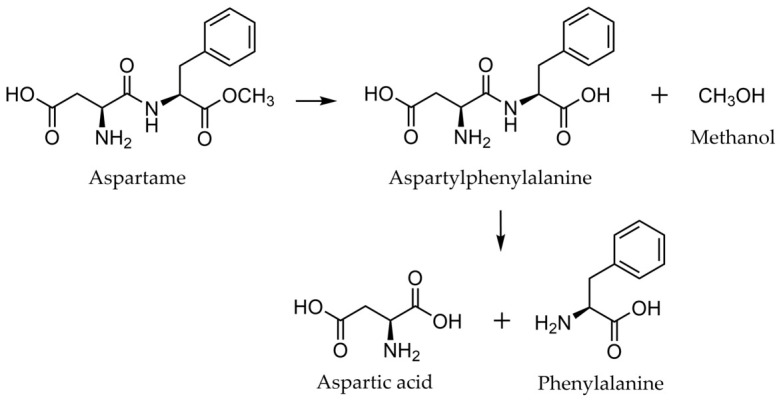
Metabolism of aspartame.

Aspartame is rapidly and completely hydrolyzed in the gastrointestinal tract after oral ingestion [11]. It is broken down to three primary metabolites: phenylalanine (≈50%), aspartic acid (≈40%), and methanol (≈10%), as presented in Figure 2. These metabolites are absorbed in the small intestine and enter normal physiological metabolic pathways rather than circulating as intact aspartame [13].

Phenylalanine is incorporated into the body’s amino acid pool and used for protein synthesis or converted to tyrosine. However, individuals with phenylketonuria are unable to adequately metabolize phenylalanine, making aspartame contraindicated for this population [28,29]. Aspartic acid functions as an excitatory neurotransmitter and is also involved in the urea cycle and energy metabolism [30]. Methanol is oxidized in the liver to formaldehyde and subsequently to formic acid, which is further metabolized to carbon dioxide and excreted [29,31]. The amount of methanol produced from aspartame consumption at typical dietary levels is generally lower than that obtained from many fruits and vegetables [29]. Importantly, aspartame itself does not reach systemic circulation in significant amounts, and its biological effects are mediated through its metabolites rather than the parent compound [32]. The safety of aspartame, its consumption and its metabolites remains controversial, as the available findings are inconsistent, underscoring the need for further well-designed studies investigating its effects [22].

#### 3.1.2. Sucralose

**Figure 3:**
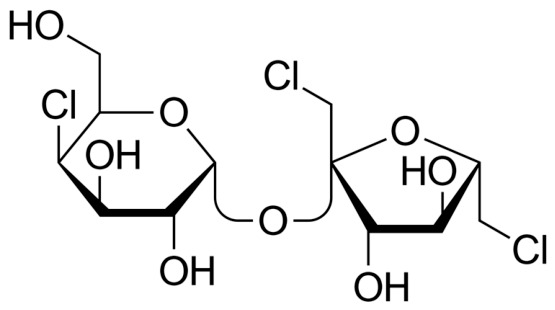
Chemical structure of Sucralose.

Sucralose (Figure 3) is a synthetic, non-caloric sweetener derived from sucrose through selective substitution of three hydroxyl groups with chlorine atoms, which confers high sweetness intensity and metabolic stability [33]. It is approximately 500–600 times sweeter than sucrose and is widely used in beverages, baked goods, dairy products, and tabletop sweeteners due to its high thermal stability and resistance to acidic conditions [34].

Following ingestion, sucralose is poorly absorbed in the gastrointestinal tract, with the majority excreted unchanged in the feces [35]. A small, absorbed fraction is eliminated via the urine, and minimal metabolism occurs in humans [35].

Despite its widespread use and regulatory approval, concerns have been raised regarding potential effects on gut microbiota, glucose metabolism, and intestinal permeability, particularly at high intake levels or with long-term consumption. Experimental studies have suggested alterations in microbial composition and enzyme activity, while human data remain limited and sometimes inconsistent [36]. As a result, ongoing research continues to evaluate the long-term safety and metabolic implications of sucralose consumption.

#### 3.1.3. Cyclamate

**Figure 4:**
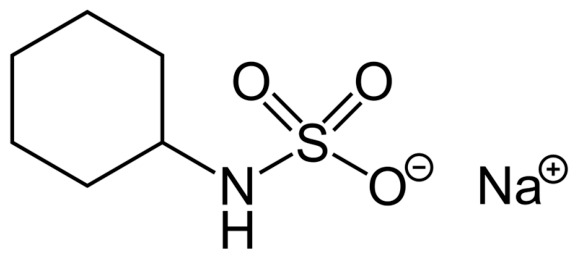
Chemical structure of Sodium Cyclamate.

Cyclamate is a non-caloric, water-soluble artificial sweetener that is 30–40 times sweeter than sucrose [37]. Due to its bitter taste, it is usually used in combination with other sweeteners such as saccharin, which leads to a synergistic increase in sweetness [38]. Despite the good solubility of the acid form of cyclamate in water (133 g/L), it can be further increased by preparing the corresponding sodium or calcium salts [37]. Sodium cyclamate (Figure 4) is widely used in low-calorie beverages and foods, as well as in the pharmaceutical industry [39,40,41,42,43].

Cyclamate is characterized by low toxicity and is considered safe, but in the gas-trointestinal tract it is metabolized to cyclohexylamine, which is known to have carcinogenic potential [44]. Although most of the consumers (about 80%) do not metabolize cyclamate to cyclohexylamine, a growing number of studies emphasize the need for restrictions on its use [45].

Potential health risks to bone health may arise from excessive consumption of sodium cyclamate. Studies reveal that even small amounts of sodium cyclamate can damage microfilaments and microtubules of osteoblasts, and its consumption may inhibit the proliferation and differentiation of osteoblasts. In addition, excessive use can lead to nervous system and liver damage. Pregnancy, young children, and the elderly are particularly susceptible. Therefore, it is necessary to be careful with its overuse and long-term consumption [43,46].

#### 3.1.4. Saccharin

Saccharin, or 1,2-benzisothiazol-3(2H)-one-1,1-dioxide, is a synthetic cyclic sulfona-mide that, along with its sodium (Na), potassium (K), and calcium (Ca) salts, is used as an artificial sweetener, approximately 300 to 500 times sweeter than sugar. [11,14]. Saccharin exhibits sweet taste through binding to multiple sites of the G-protein coupled sweet taste receptor T1R2/T1R3, with the effect being dose dependent—it elicits a sweet sensation at lower concentration, while at higher concentration it produces sweet inhibitory responses [47]. Apart from sweetness, saccharin at high concentrations exhibits bitter taste by activating the human bitter taste receptors (TAS2Rs) [38]. This explains the slightly bitter aftertaste of saccharine. For this reason, it is usually used in combination with other sweeteners [48].

One of the main advantages of saccharin is that it is poorly metabolized by host enzymes, therefore it reaches the colon unchanged and does not affect blood sugar levels [49]. Furthermore, a very small portion of consumed saccharin (15%) comes into contact with the gut bacteria, suggesting that only high doses can affect the composition of the gut microbiota [50]. However, changes in the intestinal mucosa resulting from repeated consumption of saccharin raise several concerns about its safety [49]. In the 2012 article “Etiology of Inflammatory Bowel Disease: A Unified Hypothesis”, Qin hypothesized that the increased incidence of inflammatory bowel disease is linked to impaired inactivation of digestive proteases caused by inhibition of the intestinal microbiota under the influence of food chemical agents such as saccharin and sucralose [51]. However, more research is needed to confirm these effects of saccharin on human health.

#### 3.1.5. Acesulfame Potassium (Acesulfame-K)

Acesulfame-K is the potassium salt of 6-methyl-1,2,3-oxathiazin-4(3H)-one-2,2-dioxide, a cyclic sulfonamide, structurally related to saccharin and possessing many of the same physical and chemical properties [52]. It is 200 times sweeter than sugar and is characterized by similar sweetness profile to that of saccharine—while it is half as sweet as saccharin sodium, it produces sweet taste at normal levels and bitter aftertaste at higher concentrations [52].

Acesulfame-K is widely used as a sweetener in hot drinks and syrups, due to its high solubility in water (237 g/L at 20 °C), which increases with increasing temperature [53]. It is used in combination with other sweeteners such as aspartame, sodium cyclamate and sucralose due to synergistic effects toward sweet taste enhancement and improvement of the taste profile [54].

Under acidic conditions, acesulfame-K is degraded to the low-toxicity acetylacetamide and acetoacetamide-*N*-sulfonic acid [15]. In the human body is rapidly absorbed and excreted unchanged via the kidneys in the urine, demonstrating that it is not metabolized [11]. Although approximately 99% of it is excreted unchanged in the urine, its health effects need to be thoroughly investigated, as some studies have reported its presence in amniotic fluid, umbilical cord blood, and breast milk [55,56].

#### 3.1.6. Neotame

Neotame is an *N*-alkylated aspartame derivative which is 7000–13,000 times sweeter than sugar and about 30–60 times sweeter than aspartame [16]. Regarding its flavor, neotame retains the qualities of aspartame, including the lack of bitter or metallic overtones [57]. In addition, neotame acts as a flavor enhancer, especially to the flavor of mint, and possesses a licorice-like aftertaste. Due to its peptide-like structure, it is characterized by thermal stability, slightly better than that of aspartame, which contributes to its application in cooking and baking [58,59]. However, currently, neotame is not directly available to customers for home use and is only used by food manufacturers [57].

The better stability of neotame, compared to aspartame, is due to the replacement of the -NH_3_+ group in the aspartame structure by a -NH-alkyl group (3,3-dimethylbutyl group) [60]. The dimethyl butyl group imparts the reactivity of the amino group and hinders the cyclization processes common for aspartame. Subsequently, the lack of cyclization increases the pH stability in the neutral range and the thermal stability, while the presence of dimethyl butyl group also inhibits peptidase action and the formation of phenylalanine as a breakdown product [60]. Since phenylalanine is not produced in vivo, the major metabolic pathway for neotame is hydrolysis of the methyl ester group by esterases to form de-esterified neotame and methanol in equimolar quantities [61]. Excretion of neotame and de-esterified neotame occurs through the urine and feces [61].

#### 3.1.7. Advantame

**Figure 5:**
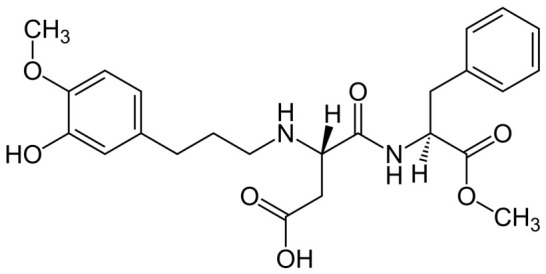
Chemical structure of advantame.

Advantame was developed as an aspartame derivative following a structure-activity relationship studies, computer modelling, and synthetic and screening program [61]. It possesses similar flavor profile to that of aspartame with no undesirable taste characteristics and is 20,000 times and 100 times sweeter than sugar and aspartame, respectively [17]. Similarly to neotame, advantame is an *N*-alkylated aspartame derivative, in which the -NH_3_+ group is replaced by a -NH-vanillin derived group, containing a 3-hydroxy-4-methoxyphenyl moiety (Figure 5) [59]. The presence of the 3-hydroxy-4-methoxyphenyl group resembles the structure of the natural sweeteners phyllodulcin and neohesperidin dihydrochalcone [17].

Pharmacokinetically, advantame is absorbed rapidly and metabolized via hydrolysis of the methyl-ester group to produce advantame-acid, which is the main plasma constituent measured after ingestion [62,63]. Advantame-acid can be subsequently metabolized to minor metabolites, including N-(3-(3-hydroxy-4-methoxy phenyl))propyl-L-aspartic acid via *N*-dealkylation and 3-[3-hydroxy-4-methoxyphenyl]-1-propylamine via amide hydrolysis [63]. All of the metabolites are excreted through the urine and feces [63].

Advantame appears to be safe and well tolerated in healthy individuals, as there are no clinically relevant changes in laboratory parameters, physical examination findings, vital signs, or ECG (electrocardiogram) after oral administration [62].

### 3.2. Regulatory Status and Acceptable Daily Intake

Synthetic sweeteners approved for use in foods and beverages are subject to rigorous safety evaluations by national and international regulatory authorities. These assessments are primarily conducted by bodies such as the FDA [9], the European Food Safety Authority (EFSA) [1], the Joint FAO/WHO Expert Committee on Food Additives (JECFA) [64], and other comparable agencies worldwide. Approval and safety assessments are based on a comprehensive review of toxicological, metabolic, and, where available, human clinical data [65,66].

A central outcome of these evaluations is the establishment of an Acceptable Daily Intake (ADI), defined as the amount of a substance that can be consumed daily over a lifetime without appreciable health risk [67]. The ADI is typically expressed in milligrams per kilogram of body weight per day (mg/kg bw/day) and is derived from the no-observed-adverse-effect level (NOAEL) identified in experimental studies, applying conservative safety factors to account for interspecies and interindividual variability [67].

Currently approved ADIs include 40 mg/kg bw/day for aspartame, 9 mg/kg bw/day for acesulfame potassium; 9 mg/kg bw/day for saccharin; and 15 mg/kg bw/day for sucralose in the European Union (EFSA) [68]; 50 mg/kg bw/day for aspartame, 15 mg/kg bw/day for acesulfame potassium, 15 mg/kg bw/day for saccharin, and 5 mg/kg bw/day for sucralose in the United States according to the FDA [9]. Neotame and advantame, due to their high sweetness potency and correspondingly low exposure levels, have higher ADIs relative to estimated intake, with neotame set at 2 mg/kg bw/day and advantame at 5 mg/kg bw/day by EFSA [68], while the ADIs set by FDA are 0.3 mg/kg bw/day for neotame and 32.8 mg/kg bw/day for advantame [9].

Dietary exposure assessments conducted by regulatory agencies generally indicate that average and high consumers remain below established ADI thresholds, including in populations with higher intake of diet beverages or sugar-free products. Nevertheless, ongoing re-evaluations are periodically undertaken as new toxicological, epidemiological, and mechanistic data emerge [69]. In recent years, particular attention has been directed toward potential long-term metabolic, neurological, and microbiome-related effects, prompting continued scientific and regulatory scrutiny despite existing approvals [70].

Overall, while synthetic sweeteners currently authorized for use are considered safe within established ADI limits, uncertainties related to chronic exposure, combined sweetener intake, and vulnerable subpopulations highlight the importance of continued monitoring and updated risk assessment based on high-quality human data.

### 3.3. Vulnerable Populations

#### 3.3.1. Pregnant Women

Gestational diabetes mellitus (GDM) is a common pregnancy complication. It is defined as the onset of glucose intolerance during the second half of gestation, or as being first recognized during pregnancy. GDM is associated with an increased risk of adverse outcomes for both mother and child [71]. Research indicates that dietary management is the most successful approach to treating GDM [72]. Consuming too many sugary drinks during pregnancy has been linked to an increased risk of gestational diabetes. To prevent excessive sugar intake, which can lead to weight gain and various chronic illnesses, many pregnant women opt for sugar-free products containing artificial sweeteners [73].

Huang et al. found that pregnant women consumed more sweeteners than non-pregnant women, and that the intake of these high-added sugars is associated with an increased risk of developing GDM [74]. The authors analyzed the association between artificial sweetener consumption during pregnancy and the incidence of GDM. The questionnaire survey included 422 pregnant women with a mean age of 32 years. The results of the study showed that pregnant women consume more artificial sweeteners than the general population. According to the study, greater use of artificial sweeteners is associated with a higher risk of GDM. Pregnant women who consumed high amounts of total artificial sweeteners were 2.6 times more likely to develop GDM than those who consumed low amounts [74].

In a longitudinal cohort study, Gjørup et al. examined the association between the consumption of artificially or sugar-sweetened beverages during pregnancy and the resulting overweight status of the offspring from birth to 18 years of age [75]. Monitoring was performed during pregnancy, childhood and adolescence. A total of 101,042 pregnant women were examined during the period 1996–2002. The authors found that daily consumption of artificially sweetened beverages during pregnancy was associated with an increased risk of being overweight during childhood and adolescence. An increase in body mass index (BMI) Z-score was observed with increased consumption of artificially sweetened beverages, indicating a dose-dependent correlation between artificial sweetener intake and the risk of being overweight in offspring [75].

Fowler et al. conducted a case–control study involving 356 children, 121 of whom acted as controls and 235 of whom were diagnosed with autism spectrum disorder (ASD) [76]. The Interactive Autism Network, an online registry of individuals with ASD and their families, was the primary source of participants. The primary objective of the study was to ascertain whether children with ASD were significantly more likely than typically developing children to have been exposed to at least one daily serving of sugar-sweetened beverages, or an equivalent intake of aspartame, during pregnancy and lactation. Evaluating whether such exposure levels were higher in offspring with any autism spectrum diagnosis than in controls was a secondary goal. The analyses were performed both separately by sex and in pooled data for each diagnostic category due to the established evidence of increased male susceptibility to early-life environmental exposures and the significantly higher incidence of autism in males—roughly four times more than in girls [76]. Boys with autism diagnoses in the study were more than three times more likely to have been regularly exposed to diet drinks or comparable amounts of aspartame from different sources regularly, either during pregnancy or through lactation. The authors conclude about possible neurological effects that require further research based on findings from the literature on increased preterm and cardiometabolic issues in children exposed to diet drinks or aspartame [76].

Methanol intake by mothers during pregnancy has been identified as a potential risk factor for the offspring. Aspartame is a common source of dietary methanol, and the main sources of dietary methanol consumption are products sweetened with aspartame [77]. The impact of aspartame’s first three phase I metabolites on brain processes has been investigated. Aspartame is transformed into methanol in the intestines, which produces formaldehyde, formate, and other poisons as well as phenylalanine, which neurons need, and aspartic acid, an excitatory neurotransmitter [78]. Aspartic acid, phenylalanine, and/or aspartame consumption can have neurotoxic consequences that include changes in neurotransmitter concentrations [79] and the induction of excitotoxic processes, both of which compromise neuronal viability and function [80]. Increased rates of neuronal death, progressive neurodegeneration, and cognitive impairments have all been connected to exposure to methanol and its main metabolite, formaldehyde. Animal models have shown changes in the gut flora after being exposed to formaldehyde, phenylalanine, and aspartame [81]. Interestingly, rats given aspartame showed a twofold increase in serum propionate levels. Increased intestinal and blood–brain barrier permeability, decreased glutathione and neurotransmitter concentrations, increased oxidative stress, excitotoxicity, and neuroinflammatory responses, as well as disturbances in mitochondrial and immune system functioning, are some of the ways that propionic acid, a short-chain fatty acid produced by the intestinal microbiome, has been linked to an increased risk of autism spectrum disorder [82,83,84].

#### 3.3.2. Children and Adolescents

Wolraich et al. conducted a nine-week, double-blind, controlled study involving two groups of children: 25 typically developing preschoolers aged 3–5 and 23 school-age children aged 6–10 whose parents identified them as being sensitive to sugar [85]. The children were placed on one of three diets: one with a high sugar content and no artificial sweeteners; one with a low sucrose content and aspartame as a sweetener; and one with a low sucrose content and saccharin (a placebo) as a sweetener. The children’s behavior and cognitive abilities were assessed every week. At the end of the study, the researchers concluded that neither aspartame nor sucrose had any significant behavioral or cognitive effects on typical preschoolers or school-age children thought to be sugar-sensitive [85].

Numerous studies have shown that regular consumption of beverages containing non-caloric sweeteners may be associated with a slight increase in BMI over time. A meta-analysis of cohort studies involving over 35,000 children (aged 2–9 years) and adolescents (aged 10–24 years) revealed that consuming an additional 355 mL of a sweetened beverage each day was associated with an average BMI increase of approximately 0.05 kg/m^2^, though this was not statistically significant compared to sugar consumption [86]. This trend is more pronounced among adolescents, boys, and cohorts with longer follow-up periods. The authors suggest that the effect may be more pronounced during periods of intense growth or following prolonged exposure [86]. An analysis of data from pediatric studies shows that the daily consumption of non-caloric sweeteners may be associated with a greater increase in BMI over the long term (e.g., after 8 years). Children who consume sweeteners daily show a greater increase in BMI over time and a higher risk of being overweight compared to children who consume them rarely or never [87]. These effects have been associated with disruption of the link between sweet taste and calorie intake: a sweet taste without calories may lead to a reduced sense of fullness and compensatory increases in food intake. Another possibility is that it increases appetite for sweet foods: regular consumption of very sweet products may maintain a preference for sweet tastes and increase total calorie intake [88,89].

#### 3.3.3. Animal Studies

Animal experiments have elucidated the possible mechanisms underlying the association between artificial sweetener consumption and metabolic alterations. These experiments have shown that artificial sweeteners can alter the composition and function of intestinal flora by destroying beneficial bacteria when they enter the gastrointestinal tract. Intestinal dysbiosis is linked to insulin resistance and systemic inflammation and is associated with poor metabolic health. Experiments have demonstrated that sucralose and aspartame modify and control the quantity and composition of the gut microbiota when ingested, and that their metabolites collectively cause metabolic problems [90]. Furthermore, certain artificial sweeteners have been shown to cause oxidative stress and inflammation, which are associated with metabolic disorders such as type 2 diabetes and insulin resistance [91]. Further research is needed to confirm these findings and provide people with guidance on better dietary practices. This should include experimental and clinical studies.

### 3.4. Studies on Metabolic and Endocrine Effects

Multiple randomized controlled trials and crossover studies have examined the metabolic and endocrine effects of artificial sweeteners in healthy individuals and patients with metabolic risk. Generally, the studies suggest that artificial sweeteners demonstrate heterogeneous and variable metabolic and endocrine effects. Studies assessing these effects are summarized in Table 1.

The most consistently evaluated artificial sweetener when it comes to metabolic effects is sucralose. In short term studies (2 to 4 weeks), no significant effects of sucralose consumption on glucose, insulin, glucagon-like peptide-1 (GLP-1), leptin or hemoglobin A1C (HbA1c) levels and insulin sensitivity were observed [92,93]. However, in longer trials (10 weeks), the administration of sucralose led to the increase in insulin levels and decrease in insulin sensitivity with no changes in glucose levels or body weight in healthy adults [94,95]. Apart from insulin, several studies also report an increase in GLP-1 levels following acute and chronic sucralose exposure [95,96]. Nevertheless, the metabolic effect of sucralose remains inconsistent.

Apart from sucralose, less consistent metabolic and endocrine effects have been observed in saccharin. In a study, evaluating the acute effects of saccharin, no significant effects on insulin secretion and blood glucose compared with water were observed in healthy young men [97]. In contrast, a cross-sectional study reported a time- and dose-dependent increase in HbA1c, fasting glucose, and oxidative stress markers (including malondialdehyde and lipid abnormalities) levels in healthy individuals and type 2 diabetes patients, following chronic consumption [98]. Saccharin intake was also associated with comparable to sucrose weight gain, assessed by a 12-week randomized controlled clinical trial as opposed to other non-nutritive sweeteners (aspartame, sucralose, and rebaudioside A) which did not significantly affect body weight [99].

The consumption of aspartame does not significantly influence fasting glucose, insulin, HbA1c, insulin sensitivity indices, or oral glucose tolerance test responses in healthy adults, as evident by multiple randomized trials lasting up to 12 weeks [92,93,100,101]. Acute and chronic aspartame intake does not affect the levels of key hormones regulating appetite, energy, and blood glucose, including GLP-1, leptin, ghrelin, and gastric inhibitory peptide [101]. In trials lasting up to 12 weeks, acesulfame-K, the effects of which are frequently evaluated in combination with aspartame, does not significantly alter fasting glucose, insulin sensitivity, insulin secretion, body weight, or dietary intake [100,102]. The aspartame derivative neotame demonstrated favorable metabolic profile in a randomized crossover trial, including lowered insulin response compared to the sucrose [103].

**Table 1:** Human studies assessing the metabolic and endocrine effects of artificial sweeteners.

Study Objective	Results	Ref.
A randomized, double-blind clinical trial evaluating the effect of sucralose and aspartame on glucose metabolism in healthy individuals.	The blood levels of glucose, insulin, active GLP-1, and leptin were similar for both groups (aspartame and sucralose) compared with the values in healthy participants, while no change in insulin sensitivity compared with the baseline values was observed.	[92]
Twelve-week randomized controlled trial comparing the effects of sucrose, aspartame, saccharin, sucralose, and rebaudioside A consumption on body weight, ingestive behaviors, and glucose tolerance in adults with overweight or obesity.	Sucrose and saccharin consumption resulted in comparable increases in body weight, while other sweeteners showed no significant effect. Sucralose reduced energy intake and produced the lowest weight gain, and glucose tolerance remained unaffected across all sweeteners.	[99]
A randomized, single-blinded, controlled study assessing the effect of regular consumption of saccharine, sucralose, aspartame, and acesulfame-K on glycemic response in healthy women.	No differences for glucose, insulin, GLP-1, or HbA_1c_ levels and insulin sensitivity at baseline or at week 4 were observed in comparison with the control group. No significant effect of sweetener consumption on body weight, body composition, and waist circumference was observed.	[93]
A randomized crossover study examining the adverse effect of soft drinks sweetened with acesulfame-K and aspartame on glucose control in normal-weight, overweight, and obese participants.	Concentrations of fasting glucose and fasting insulin, the area under the curve for an oral glucose tolerance test (OGTT) glucose and insulin, the incremental area under the curve for OGTT glucose and insulin, the homeostatic model assessment for insulin resistance, and the Matsuda index were not altered during the two-week intake of artificially sweetened drinks compared with the baseline and the control group.	[100]
A randomized controlled trial assessing the effect of daily aspartame intake for 12 weeks on glycemia, in addition to effects on appetite and body weight in lean adults.	No significant differences in glucose, insulin, resting leptin, GLP- 1, or gastric inhibitory peptide levels at baseline or week 12 were observed in the two groups. In addition, no effect on appetite and body weight was observed.	[101]
A randomized crossover clinical trial evaluating the acute effects of three soft drinks sweetened with aspartame/acesulfame-K (AAK), sucrose/stevia (SUC/STE) or sucrose on glucose and hormone responses.	Significant increase in glucose and insulin levels was observed at 30 min after sucrose-containing beverage consumption, while SUC/STE resulted in lowering the glucose levels at 60 min and sustained increase in PP levels. The AAK group and control group demonstrated no significant differences in glucose or hormone levels.	[104]
A single-blinded randomized study determining the aspartame and sucralose effect on blood glucose, insulin, c-peptide and glucagon-like GLP-1 levels in patients with type 2 diabetes.	In healthy subjects, sucralose enhances GLP-1 release and lowers blood glucose, while in type 2 diabetic patients no statistical difference in three settings for the glucose, insulin, c-peptide, and GLP-1 values were observed.	[105]
A randomized, double-blind, crossover study comparing the effects of a carbonated beverage containing aspartame and acesulfame-K consumption with those of an unsweetened carbonated beverage on insulin sensitivity and secretion in nondiabetic adults.	Consumption of beverage, sweetened with aspartame and acesulfame-K has no significant effect on insulin sensitivity and secretion, body weight, self-reported dietary consumption or physical activity in nondiabetic adults.	[102]
A randomized, parallel, double-blind, placebo-controlled trial determining the effect of acute and chronic consumption of sucralose on insulin and glucose profiles in young healthy adults.	Consumption of sucralose for 10 weeks induced increased insulin and blood glucose concentrations in the 48 mg sucralose group, increased area under the curve of insulin in both 48 and 96 mg sucralose groups, and reduced Matsuda index in the 48 mg sucralose group, suggesting that chronic consumption of sucralose can affect insulin and glucose responses in non-insulin resistant healthy young adults.	[94]
Parallel randomized clinical trial investigating the effect of sucralose consumption on concentrations of appetite-regulating hormones, including GLP- 1, ghrelin, peptide tyrosine, and leptin, and secondarily on insulin resistance in healthy, normal-weight individuals.	Sucralose consumption did not induce significant changes in concentrations of GLP-1, ghrelin, peptide tyrosine, or leptin.	[96]
A randomized double-blind placebo-controlled trial aiming to determine the effects of chronic consumption of sucralose on glycemic response, insulin secretion and sensitivity, and GLP-1 release in healthy subjects in healthy volunteers.	Active GLP-1 levels were significantly higher in the sucralose group than placebo, while acute insulin response and whole-body insulin sensitivity were lower after exposure to sucralose than placebo.	[95]
A human study assessing of the saccharin effect on insulin and blood glucose levels in healthy young men, including nine participants.	One statistically significant difference in blood glucose between sucrose and saccharin was found. Insulin secretion was significantly higher after the sucrose trial compared to the saccharin trial and water. The higher insulin levels after the saccharin trial compared to water were non-statistically significant differences.	[97]
A cross-sectional study evaluating the biochemical effects of chronic saccharin and cyclamate consumption in healthy individuals and type 2 diabetes mellitus patients.	Chronic saccharin and cyclamate consumption was associated with a time and dose-dependent effect on biochemical parameters related to metabolic functions and increased oxidative stress in both healthy and diabetic type 2 patients.	[98]
A randomized crossover trial evaluating the acute and repeated ingestive effects of biscuit formulations sweetened with neotame or stevia rebaudioside M (StRebM) vs. sucrose on appetite and endocrine responses in adults with overweight and obesity.	Appetite sensations were reduced similarly for all the formulations. Neotame and StRebM formulation showed lower postprandial insulin compared to sucrose, while glucose was lower after StRebM and not after Neotame compared to sucrose. No statistical differences in ghrelin, glucagon-like peptide 1 or pancreatic polypeptide levels were found for sweeteners and sweetness enhancers or sucrose formulations.	[103]

### 3.5. Studies on Gut Microbiota and Gastrointestinal Effects

The gut microbiome is composed of more than 1000 bacterial species, belonging to 6 phyla—*Firmicutes*, *Bacteroidetes*, *Actinobacteria*, *Proteobacteria*, *Fusobacteria*, and *Verrucomicrobia*, the most abundant of which are *Firmicutes* and *Bacteroidetes*, making up 90% of the gut microbiota [106]. *Bacteroidetes* include *Prevotella* and *Bacteroides*, while *Firmicutes* include *Bacillus*, *Lactobacillus*, *Ruminicoccus*, *Enterococcus*, and *Clostridium*. Apart from bacteria, fungi present in the gut mycobiota include *Candida*, *Saccharomyces*, *Malassezia*, and *Cladosporium* [106]. The intestinal microbiota plays a key role in food digestion, maintaining metabolic and immune homeostasis and xenobiotic and drug metabolism, so any disruptions in the gut can affect human health. Changes in the quantity and diversity of the gut microbiota may contribute to various disorders, including conditions such as inflammatory bowel disease, obesity, diabetes, cancer, and infections caused by bacteria like *Clostridium difficile* and *Escherichia coli* [107,108,109,110]. Examples of the effect of gut microflora on different diseases include the decrease in *Bifidobacterium* and *Lactobacillus* bacteria in inflammatory bowel disease and infections, increase in *Firmicutes* relative to *Bacteroidetes* in obesity, and overall reduced diversity of gut bacteria associated with diabetes, allergies, and metabolic disorders [110].

Recently, there has been increasing evidence that non-nutritive artificial sweeteners affect the composition and function of the gut microbiota, with potential implications for host metabolism and gastrointestinal health [111]. Experimental studies suggest that certain non-nutritive sweeteners can induce microbial dysbiosis, alter the production of short-chain fatty acids, and increase intestinal permeability, thereby contributing to low-grade inflammation and metabolic disturbances such as insulin resistance. However, randomized controlled trials in humans are limited. A previous study reported that regularly consuming pure aspartame or sucralose at levels consistent with high typical intake appears to have little to no impact on gut microbiota composition or the production of short-chain fatty acids [111]. Studies evaluating the effects of artificial sweeteners on the gastrointestinal tract and gut microbiota are summarized in Table 2.

### 3.6. Studies on Neurological and Behavioral Effects

#### 3.6.1. Aspartame

Once absorbed in the colon, aspartame is metabolised into phenylalanine, aspartate, and methanol. Aspartate, an excitatory neurotransmitter, acts as an *N*-methyl-D-aspartate agonist and is often found in high concentrations in the brain. Research indicates that aspartate and methanol may contribute to neurobehavioural changes by inducing oxidative stress and reducing the synthesis of dopamine and serotonin [37,117,118].

A study by Romano et al. reported several adverse effects of aspartame on the central nervous system [119]. These include headaches, mood swings, sleep disturbances, seizures, personality changes, dizziness, and vision problems [119].

Prolonged aspartame consumption has been linked to memory impairment in mice, which has been attributed to oxidative stress in their brains. This stress has been identified as a primary factor in cellular dysfunction within the hippocampus, an area characterized by high energy demands. An increased need for oxygen can disrupt mitochondrial function [120].

#### 3.6.2. Sucralose

Excessive sucralose intake has been linked to impaired memory and executive function, potentially due to alterations in the microbiome, as well as to neuroinflammation and neurotoxicity caused by metabolites of low- and no-calorie sweeteners [121]. Morales-Rio et al. conducted experimental research focusing on the peripheral and central effects of long-term consumption of nutritive and non-nutritive sweeteners [122]. Male rats were randomly divided into six groups: a control group which drank water, while the other five groups received solutions containing 10% sucrose, aspartame, sucralose, stevia or 5% xylitol. The drinking water of the treatment groups was supplemented with pure nutritive sweeteners (sucrose and xylitol) and non-nutritive sweeteners for 18 weeks. The aspartame and sucralose doses were determined using provisional daily intake levels of 4.1 and 2 mg/kg/day, respectively. The impact of prolonged sweetener consumption on memory retention in rats was assessed using the novel object recognition task. This test exploits the natural tendency of rodents to explore new objects and compare them with familiar ones [122]. The data show that long-term aspartame consumption has an adverse effect on short-term memory [123]. According to the literature, aspartame may cause oxidative stress in the brain and reduce the brain glucose availability, as well as inhibiting the brain’s production of serotonin, noradrenaline and dopamine in a dose-dependent manner. This can lead to impaired cognitive function [124]. The authors’ findings indicate that the cognitive deficit is not related to increased body weight, food or calorie intake, or glucose metabolism. Their findings corroborate existing literature suggesting a role for neuronal sweet taste receptors in synaptic function and memory acquisition [125]. It is important to note that non-nutritive sweeteners and nutritive sweeteners have different chemical structures, absorption profiles, metabolic pathways, and excretion pathways, all of which determine their impact on human health. The potential risk of cognitive changes associated with long-term non-nutritive sweeteners use necessitates further guidance on its use [122]. Studies exploring the neurological and behavioral effects of sucralose are summarized in Table 3.

**Table 3:** Human and experimental studies, investigating the neurological and behavioral effects of sucralose.

Study Objective	Results	Ref.
Longitudinal observational study investigating the correlation between the total consumption of seven sweeteners (aspartame, saccharin, acesulfame-K, erythritol, xylitol, sorbitol and tagatose) and cognitive decline in a substantial cohort of civil servants over eight years.	Participants with diabetes who regularly used low-calorie sweeteners had significantly lower verbal fluency and memory scores than participants without diabetes.	[121]
A randomized double-blind study assessing the effects of glucose and aspartame on episodic memory, word recall, and reaction times in healthy young adults.	The authors reported better results for subjects who consumed glucose-sweetened drinks than for those who consumed aspartame-sweetened drinks.	[126]
A human study examining the effects of repeated short-term use of both nutritive and non-nutritive sweeteners, such as sucralose, on CNS activity, using neuropsychological tests and quantitative electroencephalogram assessments	The participants in the sucralose group performed significantly worse in their final evaluation compared to their initial one. Their scores for overall memory, encoding memory, and executive functions all decreased after the supplementation regimen, suggesting a potentially significant effect of this sweetener on brain functions.	[127]
In vivo study in mice determining the effects of consuming the maximum allowable dose of non-nutritive sweeteners, including aspartame, stevia, and sucralose, on memory retention and on the histology of the hippocampus.	The consumption of sucralose was linked to higher theta wave activity in quantitative electroencephalography—a cognitive impairment marker.	[128]

#### 3.6.3. Saccharin

Recent data from studies of healthy individuals have demonstrated that the long-term consumption of saccharin and other low- or no-calorie sweeteners over an eight-year follow-up period is associated with an adverse impact on health [121]. According to this study, which included 12,772 participants, the consumption of saccharin and other low- or no-calorie sweeteners was associated with a faster decline in certain cognitive functions, such as verbal fluency and memory. Furthermore, the results show that higher consumption of artificially sweetened beverages is associated with an increased risk of developing dementia, Alzheimer’s disease, as well as more pronounced cognitive decline over time. Participants who consume such beverages daily are more likely to demonstrate neurodegenerative changes than individuals who rarely or never consume them. Moreover, an accelerated rate of decline in global cognition and memory was observed in patients with diabetes, while a more pronounced decline in global cognition and verbal fluency was observed in participants without diabetes [121]. The observed effect may be influenced by other health or behavioral factors, or by the fact that people with an increased metabolic risk are more likely to choose sugar-free drinks. The authors conclude that the regular consumption of low- or no-calorie sweetened beverages may be linked to an increased long-term risk of cognitive decline [121].

Experimental studies have reported that saccharin consumption leads to neurobehavioral consequences in mice. In a C57Bl/6J mouse model, McCarthy et al. observed motor hyperactivity in male mice and their offspring following exposure to saccharin [129]. Furthermore, the offspring exhibited deficits in working memory, a phenomenon not observed in the fathers. Epigenetic changes to the DNA of spermatozoa have been observed, including the hypermethylation of the promoter regions of dopamine receptor genes (D1, D4 and D5) [129]. These changes may represent a mechanism for the transgenerational transmission of behavioral phenotypes [129]. Another experimental study found that consumption of the low-calorie sweetener saccharin during the juvenile and adolescent developmental stages could impair hippocampal-dependent contextual episodic memory in a novel object in context task [130]. Deficits in spatial memory, as assessed by the Barnes maze, were also observed, but only in male rats. No anxiety-like behavior was demonstrated in either female or male animals [130].

A recent study shows that long-term consumption of beverages sweetened with saccharin leads to significant changes in cognitive functions and neurobiological mechanisms related to reward processing [131]. Using an experimental model with C57Bl/6 J adult mice, the study found that chronic intake of low doses of saccharin caused long-lasting changes in the dopaminergic system of the brain. The authors observed an imbalance in the functioning of different brain areas involved in decision-making. Specifically, increased dopamine activity has been found in the striatum, alongside reduced dopaminergic activity in the prefrontal cortex, which is responsible for cognitive control and behavioral flexibility [131]. This neurochemical imbalance is associated with a tendency towards more automatic and inflexible behavior, characterized by an impaired ability to adapt to changing conditions and a preference for immediate rewards. Furthermore, behavioral tests show that saccharin-exposed animals have impaired cognitive flexibility, an earlier tendency towards risky behavior and difficulty learning from feedback. These changes suggest a disruption to the way the brain’s reward system functions, which regulates motivation and the evaluation of the consequences of actions [131]. Additionally, the described neurocognitive effects occur despite the absence of classic metabolic disorders, such as increased body weight, altered glucose metabolism or insulin resistance. This suggests that artificial sweeteners may directly affect brain function, regardless of calorie intake [131].

In an experimental study involving Sprague-Dawley rats, the passive avoidance test revealed impaired passive learning and memory abilities after six weeks of saccharin consumption (3 mg/kg/day) [123]. Furthermore, elevated levels of brain lipid peroxidases were observed alongside increased hippocampal expression of glial fibrillary acidic protein (GFAP), a marker of astrocyte activation and inflammation [123]. Another study by Choe et al. investigated the effects of chronic saccharin consumption during the juvenile period on behavior and monoaminergic neurotransmission [132]. Juvenile mice were given a 0.2% sucralose solution from postnatal day 21 to 35. After this period, all the animals were returned to water, which allowed the assessment of both immediate and delayed effects in adulthood. Saccharin-exposed animals showed a blunted locomotor response to the amphetamine test during both the juvenile and mature stages, suggesting persistent alterations in dopaminergic sensitivity [132]. Saccharin was also found to temporarily affect reward-related behavior, where juvenile animals had a lower preference for sucrose; however, no differences were observed in adulthood [132]. The authors found that the anxiety-like behavior is modulated in an age-dependent manner: juvenile animals exposed to saccharin spend more time in the open arms of the Elevated Plus Maze, whereas adults exhibit altered patterns of environmental exploration. Measures of attention and social interaction are not significantly affected by saccharin consumption [132]. From a neurochemical perspective, the authors demonstrate that saccharin triggers region-specific alterations in dopamine and serotonin levels, as well as increasing monoamine turnover in striatal regions during the juvenile stage. In adulthood, animals exposed to saccharin exhibit elevated expression of dopamine and serotonin transporter proteins in mesocorticolimbic regions, suggesting enduring alterations in monoaminergic signaling. Notably, these effects are observed independently of caloric intake or metabolic disturbances [132].

A study was conducted in female rats to investigate whether the negative metabolic and cognitive effects caused by prolonged sugar consumption could be reversed by replacing the sugar solution with a non-caloric sweetener (saccharin) or water [133]. Female rats that were initially exposed to a 10% sucrose solution over a prolonged period developed metabolic disorders, including increased body weight, fat accumulation and impaired glucose metabolism. In addition to metabolic changes, cognitive impairment was also observed, particularly in hippocampal-dependent learning and memory tasks [133]. Interestingly, recognition memory significantly improved following the replacement of sucrose with saccharin or water, suggesting a partial recovery of brain function after the cessation of excessive caloric exposure. These findings suggest that cognitive deficits caused by long-term consumption of sucrose may be reversible if excessive calorie intake is stopped or even replaced by non-caloric sweetener [133]. An experimental study aimed to evaluate the impact of long-term saccharin consumption on the development of synaptic plasticity in the hippocampus and cerebral cortex—the neurophysiological mechanisms associated with learning and memory—in juvenile and adolescent rats [134]. The authors found that saccharin intake does not lead to significant changes in synaptic plasticity. Analysis of long-term potentiation (LTP) in the Schaffer collateral—CA1 pathway of the hippocampus did not reveal any statistically significant differences between animals that received saccharin and the control group. Similar results were observed in the somatosensory cortex, where LTP in the cortex remained unchanged regardless of sweetener intake [134]. The authors report that chronic saccharin exposure during development does not adversely affect the fundamental mechanisms of synaptic plasticity in the hippocampus or the cerebral cortex. This suggests that saccharin consumption does not impair the neural processes associated with learning and memory [134]. Studies exploring the neurological and behavioral effects of saccharin are summarized in Table 4.

**Table 4:** Human and experimental studies investigating the neurological and behavioral effects of saccharin.

Study Design	Results	Ref.
A prospective cohort study investigating the association between low-calorie and non-caloric artificial sweetener consumption, including saccharin, and the development of cognitive decline over an eight-year follow-up period. A total of 12,772 people took part in the study, and they came from a wide range of age groups, sexes, lifestyle characteristics and comorbidity backgrounds.	The consumption of saccharin and other LNCSs is found to result in a faster decline in certain cognitive functions, such as verbal fluency and memory. Furthermore, the results show that higher consumption of artificially sweetened beverages is associated with an increased risk of developing dementia, including Alzheimer’s disease, as well as more pronounced cognitive decline over time.	[121]
In vivo study in male mice investigating whether exposure to saccharin alone or saccharin combined with nicotine produces behavioral and cognitive alterations in the exposed animals and whether these phenotypes are transmitted to their offspring through epigenetic mechanisms.	Exposure of male mice to saccharin induced motor impulsivity that was transmitted to offspring, while offspring additionally showed hyperactivity and working memory deficits. These effects were associated with hypermethylation of sperm DNA, particularly in dopamine receptor gene promoter regions, suggesting epigenetic transgenerational inheritance.	[129]
In vivo study in juvenile and adolescent rats evaluating whether early-life consumption of low-calorie sweeteners (stevia, acesulfame potassium, and saccharin) affects glucose metabolism, sugar-motivated behavior, and hippocampal-dependent memory function.	Daily consumption of saccharin during the juvenile and adolescent developmental stages could impair hippocampal-dependent contextual episodic memory in male and female rats. Deficits in spatial memory were also observed, but only in male rats. No anxiety-like behavior was demonstrated in either female or male animals.	[130]
In vivo study in mice examining whether prolonged consumption of sweetened beverages, including sugar-sweetened and artificially sweetened drinks, alters cognitive function, reward processing, and decision-making behavior.	Long-term consumption of saccharin-sweetened beverages leads to significant impairment of cognitive function and changes in the neurobiological mechanisms related to reward processing. These changes are accompanied by long-lasting alterations in dopaminergic activity in various brain regions.	[131]
In vivo study in adult male rats comparing the long-term effects of artificial sweeteners, including saccharin, on brain function, oxidative stress, learning behavior, and hippocampal histology.	Saccharin impairs passive learning and memory, accompanied by increased oxidative stress and increased hippocampal expression of Glial fibrillary acidic protein. These findings suggest that long-term consumption of saccharin may have harmful effects on the cognition and hippocampal integrity of rats.	[123]
In vivo study in juvenile mice investigating the effects of excessive sucrose and saccharin intake during neurodevelopment on behavioral alterations and changes in dopamine and serotonin signaling that persist into adulthood.	Saccharin-exposed animals showed a blunted locomotor response during both the juvenile and mature stages. Anxiety-like behaviour is modulated in an age-dependent manner. Measures of attention and social interaction are not significantly affected by saccharin consumption. Animals exposed to saccharin exhibit elevated expression of dopamine and serotonin transporter proteins, suggesting enduring alterations in monoaminergic signalling.	[132]
In vivo study in female rats evaluating the behavioral and metabolic effects of switching from chronic consumption of a 10% sucrose solution to either saccharin or water, with emphasis on feeding behavior, body weight, and reward-related responses.	Chronic consumption of a 10% sucrose solution resulted in metabolic disorders and impaired learning and memory. Replacing sugar with saccharin or water significantly improved both metabolic indicators and hippocampal-dependent cognitive performance.	[133]
In vivo study in male and female rats investigating whether saccharin intake (0.1% saccharin solution for 2 h per day for three weeks) affects hippocampal and cortical synaptic plasticity, including long-term potentiation and related neural function.	No differences in the formation of long-term potentiation in the hippocampus or somatosensory cortex between male and female rats were found, suggesting that saccharin exposure during the juvenile stage did not affect synaptic plasticity in either the hippocampus or the somatosensory cortex.	[134]

#### 3.6.4. Acesulfame-K

The aforementioned study by Gonçalves et al. involved 12,772 participants and investigated the effects of saccharin and other sweeteners on cognitive function, as well as the impact of long-term exposure to acesulfame-K [121]. The analysis of the 8-year prospective study shows that higher consumption of acesulfame-K is associated with faster overall cognitive decline in terms of global cognition, memory, and verbal fluency (i.e., the ability to produce speech). This association is particularly evident in participants under 60 years of age, for whom higher intake of acesulfame-K is linked to a faster decline in cognitive performance. There is no evidence that acesulfame-K is more strongly or specifically associated with cognitive decline than other sweeteners; the data for acesulfame-K are analyzed alongside those for the others [121].

A previous study demonstrated that drinking a beverage containing a non-caloric sweetener acesulfame-K can alter the way the brain processes information about food [135]. Participants showed a stronger cognitive response to high-calorie food stimuli. They were more likely to choose such foods and reported lower satisfaction after consuming sweet foods than when they drank a sugar-sweetened beverage. It is important to note that the study did not directly measure memory, learning or attention. While it demonstrates short-term effects on the mental processing of food information and choice behavior, these cannot be extrapolated to long-term cognitive changes [135].

An experimental study investigated the effect of long-term consumption of the artificial sweetener acesulfame-K on cognitive functions in a C57Bl/6J mouse model [136]. Mice treated with acesulfame-K exhibit impaired spatial memory and reduced learning abilities in the Morris Water Maze. They also demonstrate deficits in recognition memory when assessed using the Novel object preference test. There was no change in either motor skills or anxiety, which indicates that the observed effects were specific to cognitive functions. Exposure to acesulfame-K led to the dysregulation of glycolysis and a reduction in ATP production, resulting in an impaired energy supply to neurons [136]. Furthermore, the authors observed that acesulfame-K decreased the expression of brain-derived neurotrophic factor (BDNF) in the hippocampus. BDNF is critical for synaptic plasticity, neuronal growth and survival, and is a key regulator of learning and memory [136]. The expression of tropomyosin receptor kinase B (TrkB), the BDNF receptor, was also reduced. This significantly impaired BDNF signaling, resulting in diminished support for neural networks in the hippocampus [136,137]. The authors found that acesulfame-K exposure decreased the neuroprotective Akt kinase, which is part of the PI3K/Akt pathway—a crucial mechanism for neuronal survival, protein synthesis, and energy metabolism [136,138]. Lower pathway activity makes neurons more vulnerable to stress and metabolic dysregulation. The activity of Erk1/2 (extracellular signal-regulated kinase 1/2) was also impaired by acesulfame-K. This pathway is important for synaptic plasticity, learning and memory, as well as for the transduction of BDNF signals. This data shows that consuming acesulfame-K over a long period of time leads to neurometabolic and neurosynaptic changes in hippocampal neurons, resulting in impaired memory acquisition [136].

The impact of long-term intake of the artificial sweetener acesulfame-K on brain function and cognitive performance under conditions of dietary restriction was investigated by Ibi et al. [139]. The primary goal was to evaluate whether this artificial sweetener exerts an effect on neural activity and memory processes during periods of reduced energy availability. The results show that combining acesulfame-K with a low-carbohydrate diet leads to cognitive impairment. The animals demonstrated reduced working memory, as assessed by the Y-maze test, and impaired recognition memory, as revealed by the Novel object recognition test. These changes suggest functional alterations of the hippocampus and frontal cortex—brain structures critical for learning and memory processes. A key finding of the study is that acesulfame-K intake alone does not cause cognitive impairment [139]. Adverse effects only occur when the sweetener is combined with a nutritional deficiency, suggesting that the substance’s effects are context-dependent. The authors observed a significant decrease in glucose levels in the frontal cortex, despite no significant changes in peripheral glucose. This suggests the presence of a brain-specific energy deficit, which is likely to lead to reduced neural activity and impaired synaptic plasticity. As glucose is a primary energy source for neurons, reduced availability may directly impact cognitive performance. The obtained results suggest that acesulfame-K could impair memory processes in situations involving metabolic stress or restricted energy intake by disturbing brain energy metabolism, rather than by causing direct neurotoxicity [139].

A recent study demonstrates that early exposure to acesulfame-K leads to lasting impairments in the cognitive function of rats that persist into adulthood [130]. The authors found that animals exposed to acesulfame-K during the juvenile period exhibit significant deficits in the hippocampal-dependent tasks, such as the Novel Object in Context test. In this test, the rats demonstrate an impaired ability to recognize a new object in a changed context, indicating a deficit in contextual memory. Additionally, impaired spatial learning and memory were observed in the Barnes maze task. These cognitive impairments manifest in adulthood, despite exposure to the sweetener acesulfame-K being limited to an early stage of development. This indicates that the effect on brain function is long-lasting. The authors conducted an RNA sequencing analysis of collagen-related gene pathways in the dorsal hippocampus, revealing that acesulfame-K significantly alters collagen synthesis pathways. As is well known, collagen plays a crucial role in neuronal development, including axonal guidance, glial cell differentiation and synaptogenesis [130]. In this respect, the observed changes suggest impaired synaptic organization and plasticity, which may be the underlying cause of the observed memory deficits induced by acesulfame-K. Similarly, transcriptional changes affecting genes related to glutamatergic plasticity-related pathways, synaptic function, and motivational pathways were also found in the nucleus accumbens [130]. While these changes have mainly been discussed in relation to sugar-motivated behavior, the authors also note a possible interconnection between reward systems and hippocampal circuits, which could indirectly influence cognitive processes. In addition to the observed brain changes, early exposure to acesulfame-K results in impaired glucose regulation and reduced glucose tolerance. Given the metabolic sensitivity of the hippocampus, the authors propose that metabolic dysregulation may interact with established transcriptional changes to contribute to cognitive deficits caused by acesulfame-K exposure [130]. Studies exploring the neurological and behavioral effects of acesulfame-K are summarized in Table 5.

#### 3.6.5. Neotame

There is one experimental study conducted on male albino rats to assess the effects of neotame on behavior and certain blood parameters [140]. The rats were divided into three groups: a control group that received only water; a group that received a 250 mg/kg/day dose of neotame; and a group that received aspartame for comparison purposes. The exposure period was 8 weeks. The results show that rats treated with neotame exhibit reduced locomotor activity compared to control animals, suggesting an impact on movement and general activity levels. Reduced swimming ability was observed in the swimming test, which may reflect changes in coordination or energy metabolism [140]. These results suggest that chronic exposure to neotame could negatively impact neurobehavioral functions in animals.

#### 3.6.6. Advantame

There is no data in the scientific literature on the effect of advantame on cognitive functions such as learning, memory, attention, and executive functions. Most studies published on this sweetener have focused primarily on its toxicological safety, pharmacokinetics, metabolism and potential impact on reproductive health rather than neurocognitive outcomes [141]. Available pharmacokinetic data suggest that advantame is only partially absorbed from the gastrointestinal tract [63]. A significant proportion of the administered amount is excreted either unchanged or in the form of metabolites. Due to this limited systemic exposure, the likelihood of direct effects on the central nervous system is considered low. Long-term toxicological studies in animals show no evidence of genotoxicity or carcinogenicity at doses significantly higher than those used in the food industry [142]. Although some behavioral experiments in animals have examined sweet taste preference in the presence of advantame, these studies do not assess cognitive measures such as learning or memory [143]. Furthermore, regulatory assessments by international food safety authorities lack discussion of specific cognitive effects, reflecting the limited amount of data available in this area [144]. Therefore, there is currently insufficient scientific evidence to assess the direct impact of advantame on cognitive function. Further experimental and clinical studies are required to clarify its potential impact on brain function and behavior processes.

### 3.7. Comparison with Natural and Novel Alternatives

Growing concerns regarding the long-term health effects of synthetic sweeteners have stimulated interest in natural and novel low- or no-calorie alternatives. These compounds are often perceived as safer or more “physiological” substitutes, although their health impacts are not yet fully characterized and vary substantially depending on chemical structure, metabolism, and intake level. Table 6 summarizes selected natural alternatives to artificial sweeteners, including their plant origin, sweetness potency, potential adverse effects, and applications.

Among natural high-intensity sweeteners, steviol glycosides derived from *Stevia rebaudiana* are the most widely used. Stevia and steviol glycosides, including rebaudioside A, exhibit negligible caloric value and minimal effects on glycemic and insulinemic responses [116]. Following ingestion, steviol glycosides are metabolized by the intestinal microbiota to steviol, absorbed, conjugated in the liver, and excreted primarily in the urine [145]. Regulatory agencies have established acceptable daily intakes for steviol glycosides (4 mg/kg bw/day for steviol glycosides in the EU and 12 mg/kg bw/day for rebaudioside A in the US), and current human data generally support their safety within these limits [9,68]. Rebaudioside A demonstrated favorable effects on appetite and endocrine responses in adults, showing lower postprandial insulin levels compared to sucrose, and lower glucose levels compared to neotame [103].

Monk fruit (*Siraitia grosvenorii*) sweeteners, including mogrosides (terpene glycosides), are less extensively studied, but available toxicological data suggest low risk, although robust long-term human studies are lacking [146].

Natural sweeteners, characterized by a high degree of safety, include the protein mixture thaumatin, isolated from the fruits of katemfe—*Thaumatococcus daniellii* (Benn.) Benth., Marantaceae [147,148]. The main advantages of thaumatin include its negligible caloric contribution due to its high sweetness potency and low concentration usage, a safer metabolism compared to artificial sweeteners, and low toxicity [149].

**Table 6:** Plant materials used as sweeteners.

Sweetener	Plant Origin	Sweetness Potency	Adverse Effects	Use/Remark	Ref.
Thaumatin	Fruits of *Thaumatococcus daniellii* (Benn.) Benth., Marantaceae	2000 to 3000 times sweeter than sucrose	No adverse effects were observed.	Food additive in confectionery, ice creams, chewing gum, etc.	[147]
Glycyrrhizin and other licorice root derivatives	Roots of *Glycyrrhiza glabra*, Fabaceae	About 50 times sweeter than sucrose.	Chronic excessive intake may lead to high blood pressure and low potassium levels. Inhibits the 11βHSD enzyme.	Flavoring and sweetening agent for beverages, chewing gums, candies, toothpastes, and tobacco.	[150]
Mogrosides (terpene glycosides)	Fruits of *Siraitia grosvenorii* Swingle, Cucurbitaceae	About 200–300 times sweeter than sucrose.	No adverse effects were observed.	A table-top sweetener and a non-nutritive sweetener for general use in food.	[146,151]
Steviol glycosides	Leaves of *Stevia rebaudiana* Bertoni, Asteraceae	200 to 400 times sweeter than table sugar	No adverse effects were observed.	Food industry.	[152]
Miraculin	Fruits of *Synsepalum dulcificum* Daniell., Sapotaceae	Exceeds 400,000 times the sweetness of sugar.	No adverse effects were observed.	Not approved as a standard food additive or sweetener. Commercial products containing it are generally marketed as dietary supplements rather than approved sweeteners, and availability is inconsistent.	[153,154]
Hernandulcin (sesquiterpene)	Leaves and flowers of *Lippia dulcis* Trevir., Verbenaceae	Up to 1000 times greater than sucrose.	No adverse effects were observed.	It is not used commercially as a sweetener.	[155]

Sugar alcohols (or polyols) such as sorbitol, xylitol, maltitol, and isomalt, as well as erythritol, constitute another important class of natural alternatives, approved as sweeteners and generally recognized as safe [1,68]. Erythritol is unique among polyols due to its rapid absorption in the small intestine and near-complete renal excretion in unchanged form, which limits fermentation by colonic bacteria and reduces gastrointestinal side effects commonly associated with other polyols [156]. It provides approximately 60–70% of the sweetness of sucrose while contributing negligible calories and does not significantly affect blood glucose, ghrelin and insulin levels, and demonstrates no negative effects on blood lipids, making it attractive for individuals with diabetes or metabolic disorders [157]. However, recent studies have reported associations between elevated circulating concentrations of erythritol and its downstream metabolite, erythronate, and increased cardiovascular risk in older adults, as well as an increased risk of major adverse cardiovascular events and enhanced thrombosis [158,159]. These findings warrant further investigation of the effects of erythritol on cardiovascular health.

Novel sweeteners, including rare sugars such as allulose and tagatose, have also emerged as potential alternatives to conventional synthetic sweeteners. Allulose has been shown to exert potential benefits in glycemic regulation, with a lack of negative effects on glucose levels and insulin responses [157,160]. However, data on long-term safety, habitual intake, and population-level health outcomes remain limited.

While natural and novel sweeteners may offer certain advantages over traditional synthetic sweeteners, particularly in terms of metabolic effects and consumer perception, they are not associated with risk-free consumption. The lack of long-term human studies and the limited studies assessing the health effects of these alternatives complicate direct comparisons. Therefore, further well-designed, long-term clinical studies are required to clarify the health implications of synthetic sweeteners in comparison to natural and emerging alternatives.

## 4. Conclusions

Reducing sugar intake has become a key public health objective, prompting the food industry to identify suitable alternatives to conventional sugars. Numerous artificial and natural sweeteners provide advantages in dietary management such as lowering the caloric intake. However, their long-term effects on human health continue to be investigated. Current data suggest that approved sweeteners are safe when consumed within established regulatory limits, although some uncertainties and controversies remain regarding their potential effects on metabolic health, gut microbiota, and neurobehavior. Recent investigations into the impact of artificial sweeteners on the metabolic and human gut microbiota health have yielded inconsistent findings, with some studies reporting changes in microbial composition and glucose metabolism, whereas others have found little or no effect. Moreover, the association between artificial sweeteners and neurological behavior remains unresolved, largely based on animal studies and may not represent typical human consumption. The findings of this review highlight the growing need for well-designed long-term human studies, especially in vulnerable populations. Continuous research and post-marketing surveillance are essential to further clarify the health consequences of sweetener consumption and to ensure consumers’ safety.

## Figures and Tables

**Table 2:** Studies assessing the gastrointestinal effects and the effects on gut microbiota of artificial sweeteners.

Study Objective	Results	Ref.
This study investigated the effects of sucralose and aspartame intake on gut microbiota composition in healthy adults aged 18–45 years with a body mass index (BMI) between 20 and 25.	No significant changes were observed in the median relative abundances of the dominant bacterial taxa at the family or genus level before and after exposure to either non-nutritive sweetener. The overall structure of the gut microbial community likewise remained largely unchanged. In addition, fecal short-chain fatty acid concentrations were not altered following consumption of the sweeteners.	[111]
A randomized, double-blind study assessing the short-term effects of sucralose intake on glucose regulation and gut microbiota in healthy male participants.	The consumption of high doses of sucralose for 7 days does not alter glycaemic control, insulin resistance, or gut microbiome in healthy individuals.	[112]
A study evaluating the effects of synthetic sweetener neotame on human intestinal epithelial function (Caco-2), microbiota metabolism (*Escherichia coli* and *Enterococcus faecalis*), and interactions between the intestinal epithelium and the microbiota.	At concentrations higher than 100 μM, neotame induced a significant increase in intestinal epithelial cell death. Furthermore, intestinal barrier disruption was observed at concentrations of 1–100 μM. At the same time, the model intestinal bacteria studied, *E*. *faecalis*, *Shigella*, *E*. *faecium* and *E*. *coli*, did not show any changes in the growth curve in response to neotame exposure at concentrations between 0 and 2 mM.	[113]
A cross-sectional study aimed to assess the effects of recent intake of high-intensity sweeteners (aspar-tame and acesulfame-K) on the gut microbiome in a cohort of 31 healthy adults.	Microbiome analysis revealed no notable differences in the relative abundance of bacterial taxa between consumers of aspartame or acesulfame-K and non-consumers. Bacterial diversity differed between consumers of aspartame (*p* < 0.01) and acesulfame-K (*p* = 0.03) compared to non-consumers.	[114]
A randomized, double-blind study evaluating the impact of pure saccharin on gut microbiota and glucose tolerance in healthy men and women.	Short-term supplementation with pure saccharin at maximum ADI had no effect on glucose tolerance and plasma excursions of insulin, C-peptide, glucagon or GLP-1 in healthy subjects. Daily consumption of saccharin for two weeks does not alter microbial diversity and metabolites.	[115]
A randomized-controlled trial examining the effects of non-nutritive sweeteners saccharin, sucralose, aspartame and stevia on microbiome of healthy adults.	Non-nutritive sweeteners can modify the human gut and oral microbiome in a personalized manner, leading to changes in microbial composition and function that may, in some individuals, influence glucose metabolism.	[116]

**Table 5:** Human and experimental studies investigating the neurological and behavioral effects of acesulfame-K.

Study Design	Results	Ref.
A prospective cohort study investigating the association between low-calorie and non-caloric artificial sweetener consumption, including acesulfame-K, and the development of cognitive decline over an eight-year follow-up period. A total of 12,772 people took part in the study, and they came from a wide range of age groups, sexes, lifestyle characteristics and comorbidity backgrounds.	Higher consumption of acesulfame-K is associated with faster overall cognitive decline in terms of global cognition, memory, and verbal fluency (i.e., the ability to produce speech). This association is particularly evident in participants under 60 years of age, for whom higher intake of this and other sweeteners is linked to a faster decline in cognitive performance.	[121]
In vivo study in male C57BL/6J mice investigating whether long-term exposure to acesulfame-K alters metabolic regulation, cognitive function, and neurometabolic signaling, including hippocampal neuronal activity and memory-related pathways.	Acesulfame-K -treated mice showed impaired cognitive performance, along with hippocampal metabolic dysregulation, ATP depletion, and abnormalities in BDNF/TrkB and Akt/Erk signaling pathways, suggesting that prolonged ACK intake may negatively affect neurometabolic and memory-related functions.	[136]
In vivo study in male mice investigating the long-term effects of acesulfame-K intake combined with dietary restriction (low-carbohydrate diet) for 4 weeks on cognitive and emotional brain function, including memory performance and brain glucose metabolism.	Mice on a low-carbohydrate diet with acesulfame-K exposure had impaired working memory, in addition to impaired recognition memory. The authors observed dramatically lower cortical glucose levels, suggesting that acesulfame-K may disrupt glucose transport from the blood to the frontal cortex in mice.	[139]
In vivo study in adolescent male and female rats investigating the long-term effects of habitual early-life consumption of low-calorie sweeteners, including acesulfame-K, on glucose regulation, sugar-motivated behavior, hippocampal-dependent memory, gut microbiome composition, and brain gene-expression pathways in adulthood.	Animals exposed to acesulfame-K during the juvenile period exhibited significant deficits in hippocampal-dependent tasks associated with deficits in contextual memory, impaired spatial learning and memory, as well as increased anxiety-like behavior. Acesulfame-K produced sex-dependent changes in hippocampal and nucleus accumbens gene-expression pathways associated with synaptic and collagen-related signaling.	[130]

## Data Availability

Data is contained within the article.

## Abbreviations

The following abbreviations are used in this manuscript:

ADI	Acceptable Daily Intake
ASD	Autism spectrum disorder
ATP	Adenosine triphosphate
BDNF	Brain-derived neurotrophic factor
BMI	Body mass index
CAGR	Compound annual growth rate
CNS	Central nervous system
ECG	Electrocardiogram
EFSA	European Food Safety Authority
FAO	Food and Agriculture Organization
FDA	Food and Drug Administration
FSG	Fasting blood glucose
GDM	Gestational diabetes mellitus
GFAP	Glial fibrillary acidic protein
GIP	Glucose-dependent insulinotropic peptide
GLP-1	Glucagon-like peptide-1
HbA1c	Hemoglobin A1C
JECFA	Joint FAO/WHO Expert Committee on Food Additives
LDL	Low-density lipoproteins
LTP	Long-term potentiation
MDA	Malondialdehyde
NOAEL	No-observed-adverse-effect level
OGTT	Oral glucose tolerance test
PP	Pancreatic polypeptide
SCFA	Short-chain fatty acid
TC/HDL	Total cholesterol to high-density lipoproteins ratio
TG	Triglycerides
WHO	World Health Organization
